# A Neutrophil Membrane‐Encapsulated Pd/hCeO_2_ Nanoformulation for Alleviating Age‐Dependent Perioperative Neurocognitive Disorders

**DOI:** 10.1002/advs.78033

**Published:** 2026-09-30

**Authors:** Lili Huang, Jiahui Cheng, Huan Xu, Qiang Zhang, Xiao Zhang, Ye Liu, Yizhe Zhang, Wanbing Dai, Zhi Xiong, Linghuan Guo, Lingling Xu, Wen Wang, Xin Xu, Yan Zhou, Weifeng Yu, Bo Li, Diansan Su, Daxiang Wen

**Affiliations:** ^1^ Department of Anesthesiology Renji Hospital Shanghai Jiao Tong University School of Medicine Pudong Shanghai China; ^2^ Key Laboratory of Anesthesiology (Shanghai Jiao Tong University) Ministry of Education Pudong Shanghai China; ^3^ Department of Radiology Renji Hospital Shanghai Jiao Tong University School of Medicine Pudong Shanghai China; ^4^ Department of Anesthesiology Shanghai Pulmonary Hospital Tongji University School of Medicine Yangpu Shanghai China; ^5^ Institute of Diagnostic and Interventional Radiology Shanghai Sixth People's Hospital Shanghai Jiao Tong University School of Medicine Xuhui Shanghai China; ^6^ Department of Anesthesiology The First Affiliated Hospital Zhejiang University School of Medicine Hangzhou China; ^7^ College of Health Science and Technology Shanghai Jiao Tong University School of Medicine Huangpu Shanghai China

**Keywords:** aging, glucometabolic reprogramming, microglial activation, Pd/hCeO_2_ nanozyme, perioperative neurocognitive disorder

## Abstract

Perioperative neurocognitive disorders (PND) are a prevalent and disabling complication in elderly patients after anesthesia and surgery, imposing a growing clinical burden in aging societies. Despite its high incidence, effective therapies for PND remain unavailable. Surgery‐induced neuroinflammation, characterized by excessive oxidative stress, microglial activation, and blood‐brain barrier (BBB) disruption, plays a central role in the development of PND. Here, we develop a PCN biomimetic nanozyme platform based on homologous, highly adhesive neutrophil membrane‐encapsulated Pd/hCeO_2_ nanoparticles for the treatment of age‐related PND. Benefiting from the innate inflammation‐targeting properties of neutrophil membranes, PCN efficiently traverses the compromised BBB and precisely accumulates in neuroinflammatory regions. PCN exhibits potent reactive oxygen species scavenging activity and markedly attenuates neuroinflammation in the aging brain, while preserving BBB integrity. Specifically, PCN suppresses the pro‐inflammatory and dysmetabolic state of microglia through inhibition of the glycolytic pathway and promotion of aerobic respiration, thereby protecting synaptic function and improving cognitive performance in aged PND mice. In general, this study identifies a biomimetic nanozyme strategy as a promising therapeutic approach for mitigating postoperative cognitive decline or other inflammatory damage in the aging brain.

## Graphical abstract

This study reports a neutrophil membrane‐encapsulated Pd/hCeO_2_ nanozyme (PCN) for targeted treatment of perioperative neurocognitive disorders (PND) in aged mice. PCN inhibits glycolysis‐driven microglial activation and neuroinflammation, improves mitochondrial respiration, and maintains blood‐brain barrier integrity and synaptic function, ultimately improving cognition. This work provides a biomimetic nanozyme strategy for treating age‐associated PND. The image was created in BioRender.

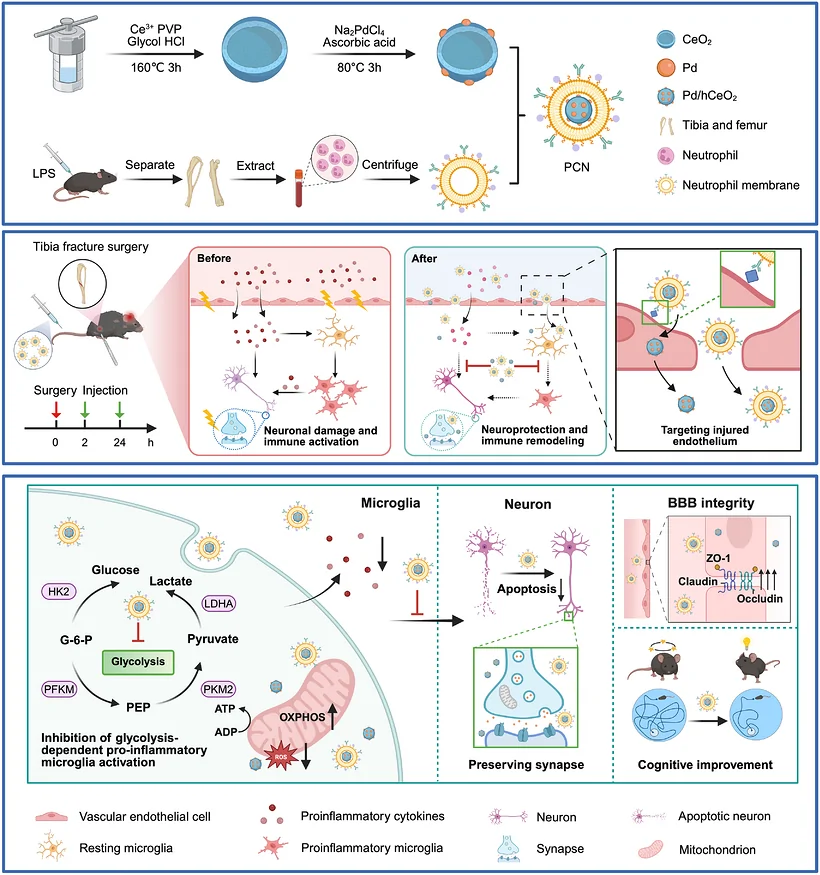

## 1 Introduction

Perioperative neurocognitive disorders (PND), encompassing postoperative delirium and longer‐lasting cognitive dysfunction, represent a major clinical challenge in aging societies, with high incidence and profound impacts on recovery, quality of life, and long‐term prognosis in elderly surgical patients [1, 2, 3, 4]. Increasing evidence indicates that surgery‐induced neuroinflammation and oxidative stress represent core pathomechanisms of PND, whereby systemic inflammatory responses propagate into the brain, disrupt BBB integrity, and trigger sustained neuroinflammatory cascades [5, 6, 7]. Current pharmacological and anesthetic interventions, including anti‐inflammatory and antioxidant strategies, have so far shown only modest and inconsistent benefits, and no disease‐modifying therapy is established for PND, especially in the aging brain [8, 9, 10, 11]. Therefore, developing multifunctional therapeutic strategies capable of simultaneously modulating key pathological processes in age‐dependent PND represents an urgent and unmet clinical need.

Nanozymes offer promising avenues for addressing neuroinflammation and oxidative damage due to their capacity for reactive oxygen species (ROS) scavenging and immune modulation [12, 13, 14]. In contrast to conventional antioxidants that are rapidly consumed or degraded in vivo, nanozymes exhibit superior stability and enzyme‐mimicking properties, enabling sustained ROS detoxification and multifunctional therapeutic effects [15, 16, 17]. Among them, CeO_2_‐based nanozymes are particularly attractive owing to the reversible Ce^3+^/Ce^4+^ redox cycle and abundant oxygen vacancies, which support continuous ROS scavenging and multiple enzyme‐like activities, together with favorable biocompatibility [18, 19, 20, 21]. Recent studies have shown that heterostructure engineering can further optimize the catalytic performance of ceria nanozymes by modulating interfacial electronic structures [22, 23]. Notably, palladium (Pd) incorporation promotes electron transfer, regulates oxygen vacancy formation, and enhances enzyme‐like activity [24, 25]. However, limited BBB penetration restricts their therapeutic efficacy in PND [26, 27, 28].

Cell membrane‐based nanotherapeutics have emerged as an effective strategy for inflammation‐associated targeting by recapitulating key biological functions of native immune cells [29, 30]. Among various membrane sources, neutrophil membranes are particularly advantageous due to their natural recruitment to inflamed tissues through adhesion molecule‐mediated interactions and chemotactic signaling pathways, enabling preferential accumulation at sites of inflammation. This enables neutrophil membrane‐encapsulated nanomaterials to target inflamed vasculature and penetrate the disrupted BBB [31, 32]. Notably, membranes derived from inflammation‐activated homologous neutrophils preserve enhanced adhesion molecule expression and chemotactic responsiveness, further improving targeting specificity to neuroinflammatory sites.

Here, we engineered a homologous neutrophil membrane‐encapsulated Pd/hCeO_2_ nanozyme (PCN) that harnesses neutrophil‐mediated inflammatory recruitment to traverse the compromised BBB and preferentially accumulate in neuroinflammatory regions. To enhance targeting efficiency, neutrophil membranes were obtained following in vivo inflammatory stimulation, thereby improving adhesion to inflamed endothelium (Figure 1A). Benefiting from both the inflammation‐targeting property and the potent ROS‐scavenging activity, PCN exhibits excellent biocompatibility and robust accumulation in inflamed cerebrovascular regions, thereby initiating a cascade of neuroprotective effects (Figure 1B). Mechanistically, PCN attenuates microglial polarization toward a glycolysis‐biased pro‐inflammatory phenotype by suppressing glycolysis while enhancing mitochondrial oxidative phosphorylation (OXPHOS). Consequently, this metabolic reprogramming is accompanied by reduced neuronal apoptosis, preservation of synaptic structure and function and BBB integrity, and ultimately improvement of postoperative cognitive outcomes in aged mice (Figure 1C). Collectively, this work highlights a biomimetic nanozyme strategy as a promising therapeutic approach for surgery‐induced cognitive decline in the aging brain.

**FIGURE 1:**
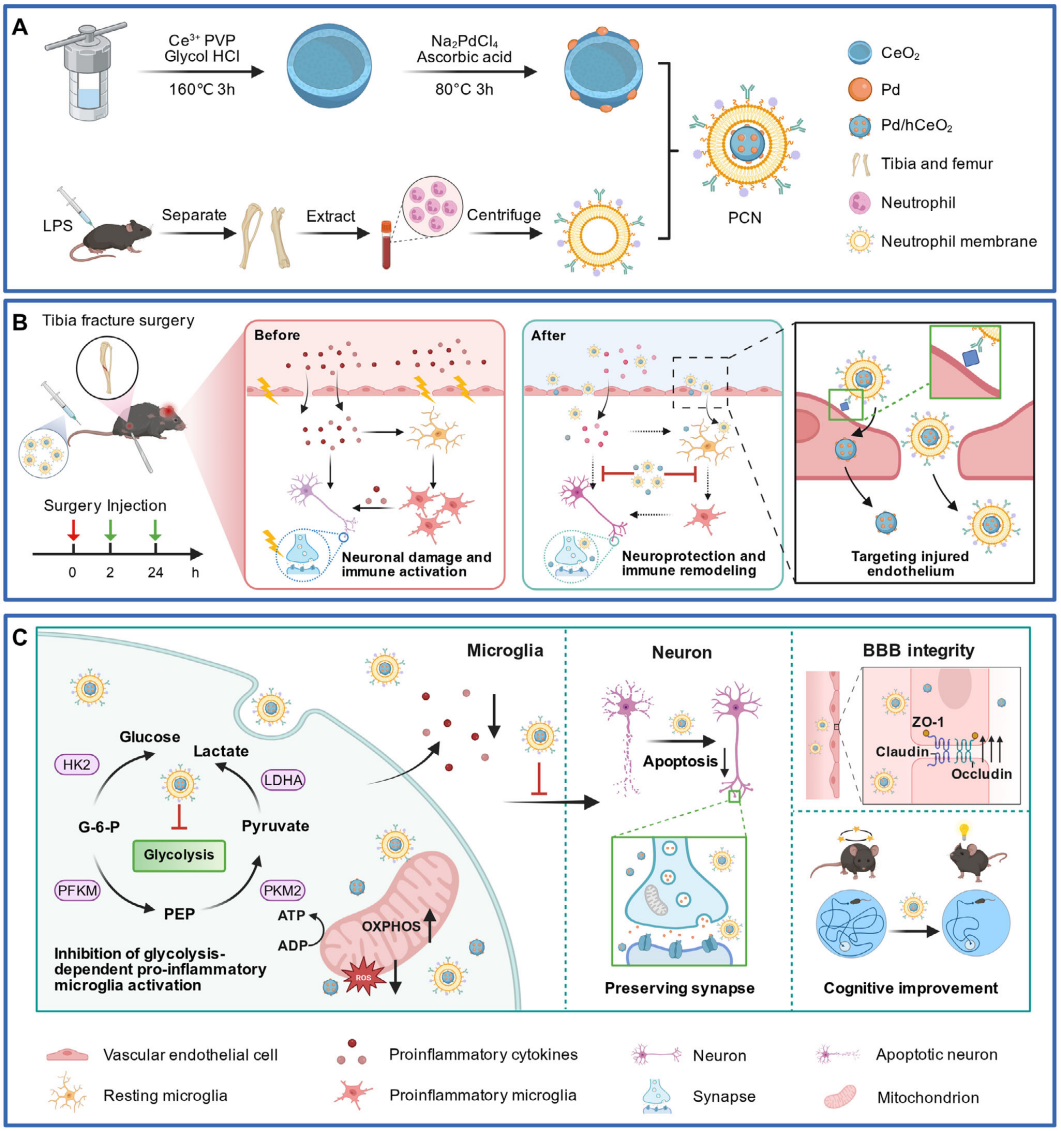
Design rationale and therapeutic mechanism of PCN for aged PND. (A) Schematic illustration of the construction of a homologous neutrophil membrane‐camouflaged Pd/hCeO_2_ nanoplatform (PCN). (B) Following intravenous administration, PCN leverages innate neutrophil inflammatory chemotaxis to traverse the disrupted BBB and preferentially accumulate in inflamed cerebrovascular and parenchymal regions. (C) In the aging brain, PCN modulates microglial inflammatory and metabolic states, attenuates oxidative stress and neuroinflammation, preserves synaptic structure and BBB integrity, and ultimately improves cognitive function in aged PND mice. Figure 1 was created in BioRender. Huang, L. (2026) https://BioRender.com/u8g59kf.

## 2 Results and Discussion

### 2.1 Design, Characterization, and PND Brain‐Targeting Delivery of the PCN Nanosystem

To obtain nanozyme drugs with high catalytic activities, hollow cerium oxide (hCeO_2_) nanospheres were first synthesized via a controlled templating strategy [22], followed by temperature‐regulated reductive deposition of palladium nanoclusters to generate Pd/hCeO_2_ nanoparticles (NPs). Transmission electron microscopy (TEM) revealed uniform hollow spherical architectures with an average diameter of approximately 100 nm, in which Pd nanodomains were embedded within the hCeO_2_ shell, forming a heterogeneous interface (Figure 2A–C). High‐angle annular dark‐field scanning transmission electron microscopy (HAADF‐STEM) and elemental mapping confirmed the co‐localization of Pd, Ce, C, N, and O, verifying successful heterostructure construction (Figure 2D). X‐ray diffraction (XRD) and x‐ray photoelectron spectroscopy (XPS) analyses confirmed effective Pd incorporation and revealed a significantly increased Ce^3^^+^/Ce^4^^+^ ratio in Pd/hCeO_2_ compared with pristine hCeO_2_ (Figure 2F, G), indicating enriched oxygen vacancies and enhanced redox activity relevant to ROS scavenging, with reference to previous reports on ceria‐based nanozymes [33]. These features are well suited to counteract oxidative stress, a central driver of postoperative neuroinflammation.

**FIGURE 2:**
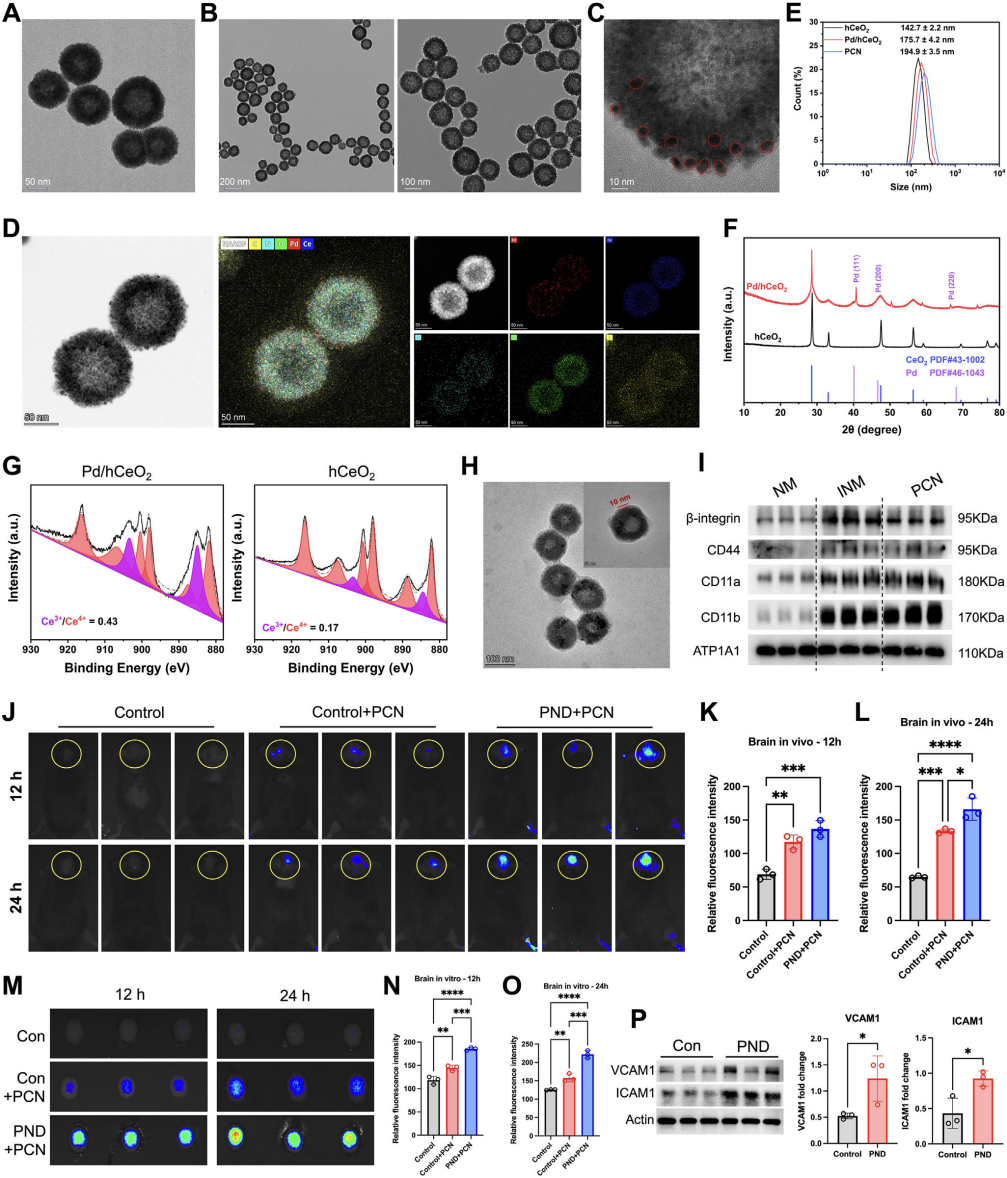
Construction, characterization, and aged PND brain‐targeting performance of PCN. (A–C) TEM images showing the hollow spherical morphology of hCeO_2_ and the heterogeneous surface architecture of Pd/hCeO_2_ nanoparticles with Pd nanoclusters embedded in the hCeO_2_ shell. Scale bars, 50 nm (A), 200, 100 nm (B), and 10 nm (C). (D) HAADF‐STEM image and corresponding elemental mappings confirming the co‐localization of Pd, Ce, and O in the Pd/hCeO_2_ heterostructures. Scale bars, 50 nm. (E) DLS analysis showing the hydrodynamic size distributions of hCeO_2_, Pd/hCeO_2_, and PCN, with a moderate size increase after membrane coating. (F) XRD patterns of hCeO_2_ and Pd/hCeO_2_, confirming Pd incorporation into the CeO_2_ lattice. (G) XPS spectra of hCeO_2_ and Pd/hCeO_2_, revealing altered cerium valence states associated with enhanced antioxidant activity. (H) TEM image of Pd/hCeO_2_@highly adhesive neutrophil membrane (PCN), with membrane‐like translucent structures encapsulated on the surface. Scale bars, 100 nm. (I) Western blot analysis showing the expression of key adhesion proteins (β2‐integrin, CD11a, CD11b, and CD44) on PCN, neutrophil membranes with inflammatory stimulation (INM), and neutrophil membranes without inflammatory stimulation (NM) (*n* = 3). (J–L) In vivo fluorescence imaging and quantitative analysis showing preferential accumulation of PCN in the brain and peripheral surgical sites of aged PND mice at 12 and 24 h after intravenous administration (*n* = 3). (M–O) Ex vivo fluorescence imaging of brain tissues further confirming enhanced cerebral accumulation of PCN in PND mice compared with age‐matched control mice (*n* = 3). (P) Western blot analysis of vascular adhesion molecules VCAM‐1 and ICAM‐1 in hippocampus from aged PND mice (*n* = 3). Data are presented as means ± standard deviation (SD). Statistical significance was determined using a two‐tailed unpaired Student's *t*‐test for comparisons between two groups and one‐way ANOVA followed by Tukey's post hoc test for multiple‐group comparisons. ^*^ *p* <0.05, ^**^ *p* <0.01, ^***^ *p* <0.001, and ^****^ *p* <0.0001.

Despite favorable biocatalytic properties, effective application in PND critically depends on overcoming biological delivery barriers, particularly the BBB. The impaired BBB integrity in the aging brain of PND provides an outlet for the design of inflammation‐specific targeting regimens. Neutrophils are rapidly mobilized following surgical stress and preferentially adhere to activated cerebrovascular endothelium, contributing to BBB disruption and postoperative neuroinflammation [34, 35, 36]. Inspired by these innate chemotactic properties and to further reduce the immunogenicity of the envisioned nanomedicine, highly adhesive neutrophil membranes were isolated from lipopolysaccharide (LPS)‐stimulated littermate mouse bone marrow and coated onto Pd/hCeO_2_ NPs via an extrusion‐assisted assembly, yielding a homology‐modified PCN (Pd/hCeO_2_@highly adhesive neutrophil membranes) biomimetic nanozyme system. TEM revealed that the PCN surface exhibits a clear membrane‐like structure (Figure 2H). Western blot analysis demonstrated that the expression levels of key adhesion proteins (including β2 integrin, CD11a, CD11b, and CD44) in PCN were similar to those in inflammation‐stimulated neutrophil membranes prior to encapsulation and were significantly higher than those in non‐inflammation‐stimulated neutrophil membranes (Figure 2I and Figure S1), indicating that PCN retained the molecular determinants required for endothelial recognition and sensitive inflammation‐guided targeting. Dynamic light scattering (DLS) and ζ‐potential analyses showed a modest increase in hydrodynamic diameter and a surface charge shift after membrane coating (Figure 2E and Figure S2).

Following successful nanomedicine construction, we next assessed whether PCN exhibited in vivo targeting properties in aged PND mice. In vivo fluorescence imaging demonstrated that PCN preferentially accumulated at the surgical site and in the brain of PND mice compared with age‐matched control mice, with detectable signals persisting beyond 24 h after administration (Figure 2J–L). Ex vivo imaging further confirmed the enhanced PCN enrichment in the brains of PND mice (Figure 2M–O). Interestingly, the pronounced accumulation of PCN at the tibial fracture site suggests that PCN can also target the peripheral inflammatory source induced by surgical trauma. Given that the surgical site serves as an important inflammatory niche after trauma [5, 37], PCN enrichment at the fracture site may help reduce peripheral inflammatory signals and limit their subsequent impact on the brain. The biodistribution of PCN in other major organs, including the heart, liver, spleen, lungs, and kidneys, was further evaluated at 12 and 24 h after administration (Figures S3 and S4). The preferential enrichment of PCN in inflammation‐associated tissues was attributable to the inflammation‐homing properties conferred by the neutrophil membrane coating. Furthermore, elevated expression of vascular adhesion molecule‐1 (VCAM‐1) and intercellular adhesion molecule‐1 (ICAM‐1) was observed in PND mice (Figure 2P), indicating endothelial activation following surgery‐induced inflammation and BBB impairment. These findings provide a molecular basis for the endothelial adhesion and inflammatory targeting capability of PCN mediated by homologous neutrophil membranes. At the same time, given its targeted distribution in both peripheral and central inflammatory sites, PCN is not merely a drug delivery system for the brain; it also has the potential to serve as a targeted modulator for systemic inflammatory diseases.

### 2.2 DFT Calculation of Nanozyme Activities and ROS‐Scavenging Capability of PCN

Given the central role of oxidative stress in driving postoperative neuroinflammation and cognitive impairment, we next investigated the ROS scavenging enzyme‐mimetic mechanisms of PCN. To this end, density functional theory (DFT) calculations were performed to compare the catalytic activities of pristine hCeO_2_ and Pd/hCeO_2_. Guided by TEM and XPS analyses, a Pd/hCeO_2_ surface model was constructed (Figure 3). Charge density difference (CDD) mapping revealed pronounced electron transfer from Pd nanoclusters to the CeO_2_ support (2.58 |e|), resulting in increased electron density at surface oxygen species and facilitating catalytic activation (Figure 3B, C).

**FIGURE 3:**
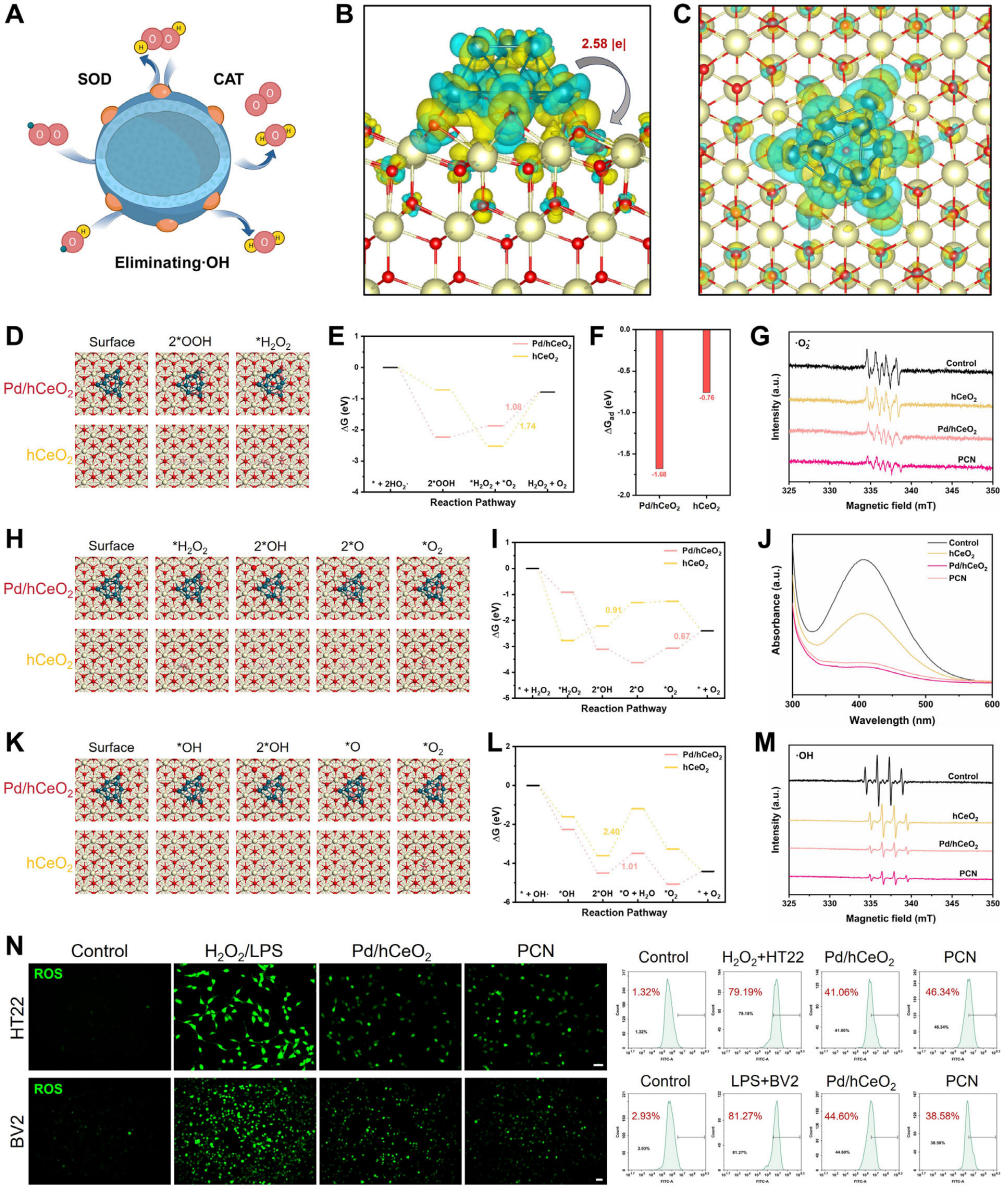
Density functional theory analysis and in vitro validation of the enzyme‐mimetic activities of PCN. (A) Schematic representation of the multiple enzyme‐mimicking activities of Pd/hCeO_2_. Figure 3A was created in BioRender. Huang, L. (2026) https://BioRender.com/1147dq7. (B, C) Charge density difference mapping showing pronounced electron transfer from Pd to the CeO_2_ support, with increased surface oxygen electron density and catalytic activity. (D, E) Calculated reaction energy profiles for the SOD‐like catalytic pathway on hCeO_2_ and Pd/hCeO_2_, highlighting reduced activation barriers on Pd/hCeO_2_. (F) Adsorption energies of •O_2_^−^ on hCeO_2_ and Pd/hCeO_2_ surfaces, showing strengthened superoxide adsorption after Pd decoration. (G) ESR spectra of •O_2_^−^ scavenging using 5‐dimethyl‐1‐pyrroline‐N‐oxide (DMPO) as the spin trap after treatment with hCeO_2_, Pd/hCeO_2_, or PCN. (H, I) Reaction energy profiles of CAT‐like catalytic pathway on hCeO_2_ and Pd/hCeO_2_. (J) UV–vis assessment of H_2_O_2_ decomposition for evaluating CAT‐like activity of different nanoparticles using a CAT assay kit. (K, L) Reaction energy profiles of the •OH elimination pathway on hCeO_2_ and Pd/hCeO_2_, demonstrating broadly reduced energy barriers following Pd decoration. (M) ESR spectra for •OH scavenging using DMPO as the spin trap in the presence of hCeO_2_, Pd/hCeO_2_, or PCN. (N) Representative DCFH‐DA fluorescence staining images (left) and flow cytometry (right) analysis of intracellular ROS levels in HT22 neurons and BV2 microglia after oxidative or inflammatory stimulation. Data are presented as means ± SD (*n* = 3 independent experiments).

Across multiple reaction pathways, Pd decoration consistently lowered the energy barriers associated with ROS conversion. In the superoxide dismutase (SOD)‐like reaction, Pd/hCeO_2_ exhibited substantially reduced activation energies compared with hCeO_2_, particularly at the rate‐determining step, indicating a more favorable catalytic pathway (Figure 3D, E). In parallel, Pd incorporation markedly strengthened superoxide anion (•O_2_^−^) adsorption, further promoting SOD‐like conversion (Figure 3F). Similar barrier reductions were observed for catalase (CAT)‐like activity (Figure 3H, I) and hydroxyl radical (•OH) elimination (Figure 3K, L), underscoring the broad catalytic enhancement conferred by Pd decoration across distinct ROS‐scavenging processes. Collectively, these theoretical analyses indicate that Pd decoration enhances the redox catalytic performance of hCeO_2_ by modulating surface reaction energetics through electron transfer‐mediated mechanisms. Importantly, these computational predictions were well supported by experimental enzyme‐mimetic activity assays. Electron spin resonance (ESR) spectroscopy revealed pronounced attenuation of •OH and •O_2_^−^ signals following treatment with Pd/hCeO_2_ or PCN, accompanied by a marked reduction in H_2_O_2_‐associated absorbance, demonstrating robust multi‐enzyme–mimetic activities (Figure 3G, J, M). Notably, the ROS‐scavenging capacity of PCN closely matched that of Pd/hCeO_2_ and substantially exceeded hCeO_2_, indicating that neutrophil membrane cloaking preserves the intrinsic catalytic activity of the Pd/hCeO_2_ core. The elevated Ce^3+^/Ce^4+^ ratio within the nanozyme further contributes to its enhanced redox reactivity.

Given these potent enzyme‐mimetic properties, we next evaluated the ability of Pd/hCeO_2_ and PCN to attenuate oxidative stress in neural cell models. We first assessed the cellular uptake behavior of PCN in HT22 neurons and BV2 microglia, which showed a time‐dependent increase in uptake efficiency (Figure S5). To confirm the role of Pd incorporation, we compared hCeO_2_ and Pd/hCeO_2_ in LPS‐stimulated BV2 cells and found that Pd/hCeO_2_ more effectively suppressed intracellular ROS levels than hCeO_2_ (Figure S6), indicating enhanced antioxidant activity. Subsequently, HT22 neurons and BV2 microglia were exposed to H_2_O_2_ or LPS, respectively, to induce oxidative and inflammatory stress. Intracellular ROS levels were quantified using 2′,7′‐dichlorofluorescin diacetate (DCFH‐DA) staining. Both Pd/hCeO_2_ and PCN markedly reduced ROS accumulation in HT22 neurons and BV2 microglia (Figure 3N), confirming effective intracellular antioxidant activity under pathological stress conditions.

### 2.3 PCN Alleviates Postoperative Cognitive Deficits and Synaptic Dysfunction in Aged PND Mice

An established PND model was induced in aged mice by tibial fracture with intramedullary fixation under isoflurane anesthesia, followed by intravenous administration of Pd/hCeO_2_ or PCN at 2 and 24 h after surgery (Figure 4A). Open‐field test (OFT) showed no significant differences in locomotor activity or anxiety‐like behavior among groups, excluding motor dysfunction or anxiety as confounding factors (Figure 4B, C). Spatial learning and memory were assessed using the Morris water maze (MWM). During the hidden‐platform training phase, PND mice displayed prolonged escape latency relative to controls, while PCN‐treated mice showed significant reductions in escape latency, indicating improved spatial learning (Figure 4D, E). Importantly, swimming speed during the training phase did not differ among groups, indicating that changes in escape latency were not attributable to alterations in locomotor performance (Figure S7A). In the probe test, PND mice spent less time in the target quadrant and exhibited fewer platform crossings than controls, whereas PCN treatment significantly restored these impairments, suggesting enhanced spatial learning ability (Figure 4F and Figure S7B). The novel object recognition (NOR) test was performed to evaluate recognition memory. PND mice showed reduced exploration of the novel object and a lower discrimination index compared to controls. The discrimination index was significantly increased in PCN‐treated mice compared with the PND group, whereas Pd/hCeO_2_ treatment showed only a nonsignificant trend toward improvement, suggesting a cognitive benefit conferred by neutrophil membrane modification (Figure 4G, H).

**FIGURE 4:**
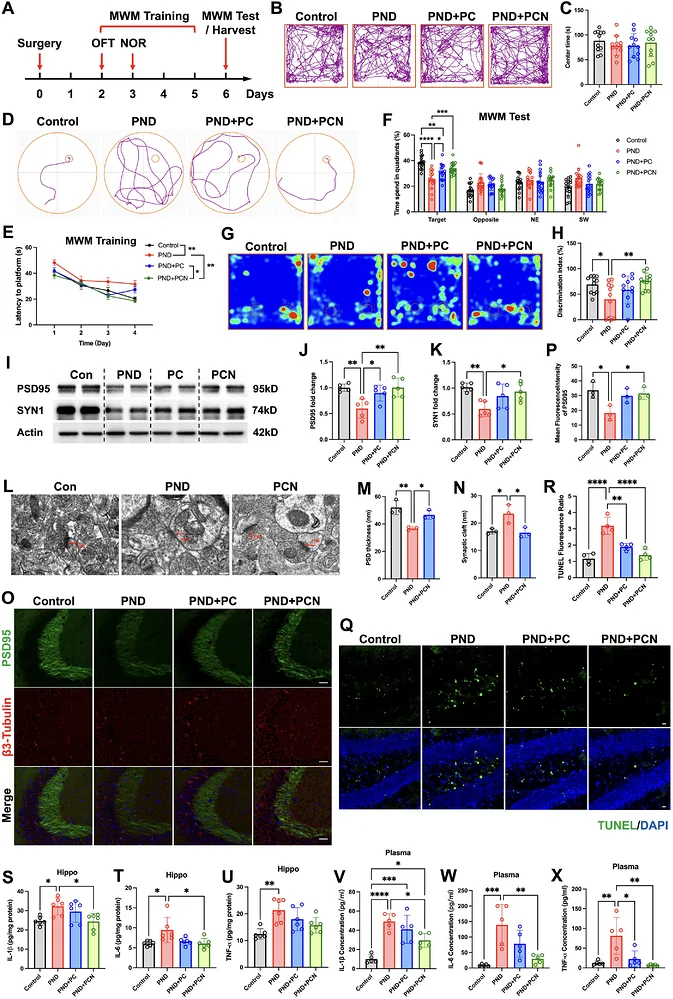
PCN alleviates postoperative cognitive deficits, neuronal apoptosis, and neuroinflammation and preserves synaptic integrity in aged PND mice. (A) Schematic illustration of the experimental timeline for PND induction by tibial fracture and fixation surgery, subsequent intravenous administration of Pd/hCeO_2_ (PC) or PCN at 2 and 24 h post‐surgery, and behavioral assessments. (B, C) OFT showing comparable locomotor activity and center‐zone exploration time among groups (*n* = 11). (D) Representative swimming trajectories of mice in the MWM test. (E) MWM testing for spatial learning curves during training in aged mice, measured as latency to find the hidden platform (*n* = 16). (F) MWM probe test showing percent time spent in different quadrants of the four groups (*n* = 16). (G) Representative activity heat maps of mice in the NOR test. (H) NOR test showing discrimination index (*n* = 11). (I–K) Western blot analysis and quantification of synaptic proteins PSD95 and SYN1 in the hippocampus (*n* = 5). (L–N) TEM images depicting hippocampal synaptic ultrastructure and statistics of PSD95 thickness and synaptic cleft (*n* = 3). Scale bars, 200 nm. (O, P) Representative immunofluorescence images and mean fluorescence intensity of PSD95 in the hippocampus (*n* = 3). Scale bars, 50 µm. (Q, R) TUNEL staining and quantification showing neuronal apoptosis in the hippocampus (*n* = 4). Scale bars, 50 µm. (S–U) Levels of IL‐1β, IL‐6, and TNF‐α in hippocampal tissues at 48 h post‐surgery (*n* = 6). (V–X) Levels of pro‐inflammatory cytokines IL‐1β, IL‐6 and TNF‐α in plasma at 48 h post‐surgery (*n* = 5). All data are presented as mean ± SD, except for MWM training latency data, which are shown as mean ± standard error of the mean (SEM). Escape latency during the MWM training phase was analyzed using two‐way repeated measures ANOVA followed by Tukey's post hoc test. Other data were analyzed using one‐way ANOVA followed by Tukey's multiple comparisons test. ^*^ *p* <0.05, ^**^ *p* <0.01, ^***^ *p* <0.001, and ^****^ *p* <0.0001.

Consistent with in vitro findings, dihydroethidium (DHE) staining of hippocampal sections showed increased ROS production in aged PND mice, whereas PCN treatment markedly reduced hippocampal DHE fluorescence intensity, indicating effective attenuation of surgery‐induced oxidative stress in vivo (Figure S8). Given that hippocampus‐dependent learning and memory critically rely on synaptic plasticity, and synaptic dysfunction is a key pathological substrate underlying cognitive impairment in PND [6, 38], we next examined whether the cognitive benefits conferred by PCN were associated with preservation of synaptic structure and function. Western blot and immunofluorescence analyses revealed that the expression of key synaptic proteins, including postsynaptic density protein 95 (PSD95) and synapsin‐1 (SYN1), was markedly reduced in the hippocampus of PND mice compared with controls. PCN treatment significantly restored the levels of both PSD95 and SYN1, whereas Pd/hCeO_2_ produced a weaker effect (Figure 4I–K, O, P). Consistently, morphological detection revealed substantial improvements in synaptic ultrastructure in the PCN‐treated group, characterized by increased postsynaptic density thickness and reduced synaptic cleft width compared with the PND group (Figure 4L–N). In vitro findings further support that oxidative stress–induced reductions in PSD95 and synaptophysin can be reversed by Pd/hCeO_2_ or PCN treatment in HT22 neurons (Figure S9). Beyond inflammatory regulation, microglia regulate synaptic homeostasis through pruning and phagocytosis [39]. Recent evidence indicates that complement‐dependent microglial phagocytosis contributes to anesthesia/surgery‐induced synaptic loss and cognitive impairment [40]. Therefore, PCN‐mediated suppression of microglial activation and preservation of synaptic integrity may also involve modulation of microglia–synapse interactions, which requires further investigation.

Since synaptic impairment in PND frequently coincides with neuronal damage, we further detected whether PCN treatment could also mitigate neuronal apoptosis in the hippocampus. Neuronal apoptosis was markedly increased in the hippocampus of aged PND mice, as evidenced by elevated numbers of terminal deoxynucleotidyl transferase–mediated dUTP nick‐end labeling (TUNEL)‐positive cells. PCN intervention efficiently decreased the surgery‐induced neuronal loss, and Pd/hCeO_2_ exerted a weaker effect (Figure 4Q, R). Consistent with these in vivo findings, oxidative stress induced pronounced apoptosis in HT22 cells, which was markedly attenuated by both Pd/hCeO_2_ and PCN treatment (Figure S10C, D). Moreover, JC‐1 staining showed that H_2_O_2_ exposure caused mitochondrial membrane potential loss in HT22 cells, whereas PCN treatment partially restored mitochondrial membrane potential, further supporting its mitochondrial protective effect under oxidative stress (Figure S11). In addition, PCN exerted a dose‐dependent cytoprotective effect (Figure S10B). These results support the robust anti‐apoptotic and neuroprotective efficacy of PCN.

As neuroinflammation‐driven synaptic dysfunction represents a central pathological feature of PND, we next quantified inflammatory cytokine levels in both peripheral blood and hippocampal tissues. At 48 h post‐surgery, levels of interleukin‐1β (IL‐1β), interleukin‐6 (IL‐6), and tumor necrosis factor‐alpha (TNF‐α) were significantly elevated in PND mice. In contrast, PCN treatment robustly suppressed the postoperative increase of these cytokines, while Pd/hCeO_2_ exerted a comparatively weaker anti‐inflammatory effect (Figure 4S–X). Together, these findings demonstrate that PCN can preserve synaptic integrity, attenuate neuronal apoptosis and inflammation, and inhibit peripherally initiated pro‐inflammatory signaling. This coordinated neuroprotective and anti‐inflammatory action provides a mechanistic basis for the observed improvement in cognitive function following PCN treatment in aged PND mice.

### 2.4 Transcriptomic Profiling Reveals PCN‐Mediated Regulation of Inflammatory and Immune Networks in Aged PND Mice

To elucidate the molecular mechanisms underlying the therapeutic effects of PCN in age‐associated PND, bulk RNA sequencing was performed on hippocampal tissues from control, PND, and PCN‐treated aged mice. Principal component analysis (PCA) revealed a clear separation between PND and control groups, whereas PCN‐treated samples clustered closer to controls, indicating partial reversal of surgery‐induced transcriptional alterations (Figure 5A). Differential expression analysis identified 553 differentially expressed genes (DEGs) between PND and control groups (366 upregulated and 187 downregulated genes) and 473 DEGs between PCN‐treated and PND groups (158 upregulated and 315 downregulated genes) (Figure 5B, C). Venn diagram further illustrated overlapping genes between PND‐associated and PCN‐responsive transcriptional changes, suggesting that PCN modulated a subset of surgery‐induced molecular alterations (Figure 5D).

**FIGURE 5:**
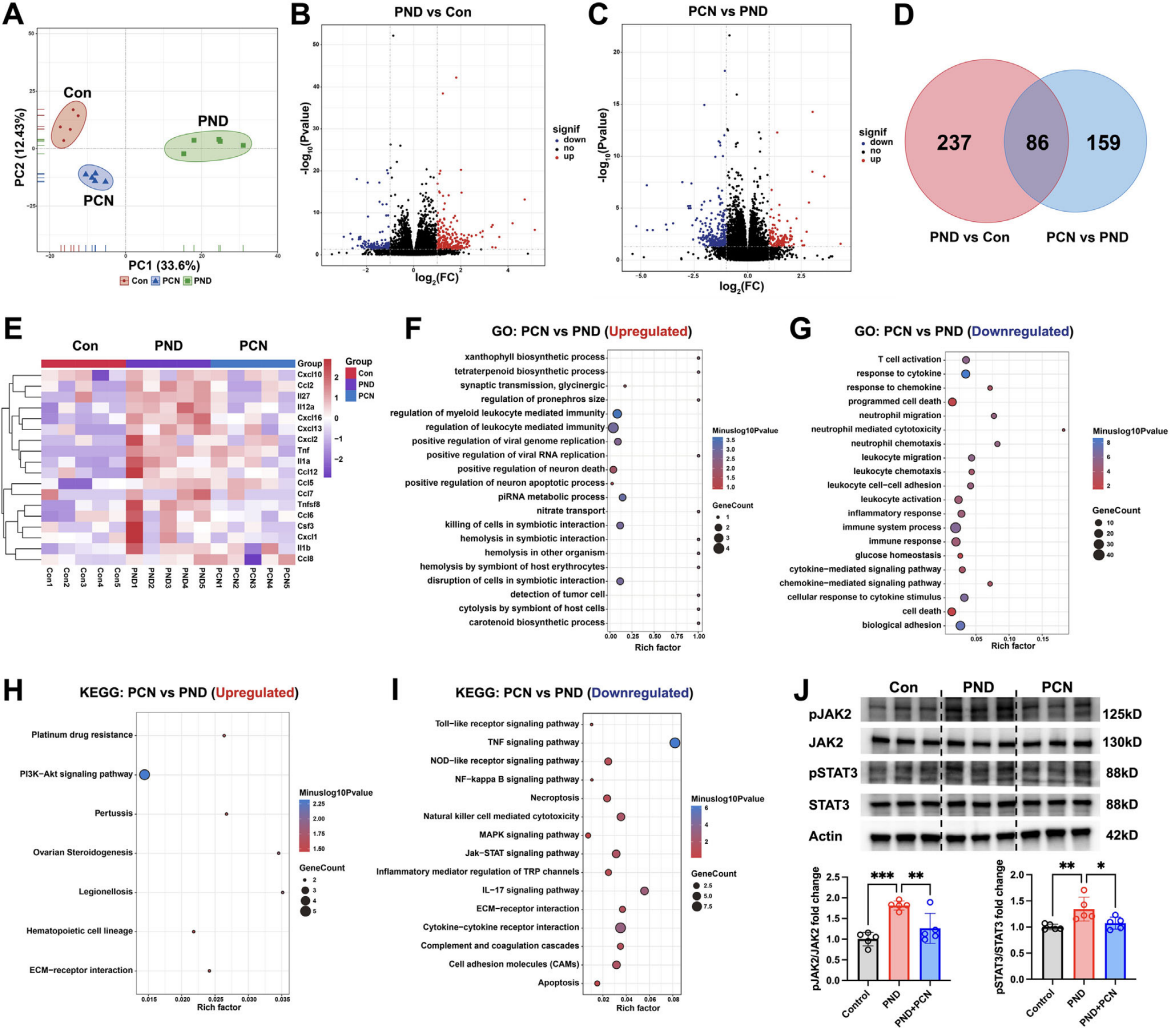
Transcriptomic profiling of hippocampal tissues following PCN treatment in aged PND mice. (A) Principal component analysis showing the global transcriptional profiles of control, PND, and PCN‐treated mice (*n* = 5). (B, C) Volcano plots illustrating differentially expressed genes (DEGs) of the PND vs. control and PCN vs. PND groups. (D) Venn diagram showing the overlap of DEGs among the three groups. (E) Heatmap showing the mRNA expression patterns of representative pro‐inflammatory and immune‐related DEGs identified from the transcriptomic analysis across the Con, PND, and PCN groups. (F, G) Gene Ontology (GO) analysis showing biological processes enriched by upregulated (F) and downregulated (G) DEGs in the PCN vs. PND comparison. (H, I) Kyoto Encyclopedia of Genes and Genomes (KEGG) pathway enrichment analysis showing pathways enriched by upregulated (H) and downregulated (I) DEGs in the PCN vs. PND comparison. (J) Western blot analysis of pJAK2, JAK2, pSTAT3, and STAT3 in hippocampal tissues from the indicated groups. Data are presented as means ± SD in Western blot analysis. Statistical significance was determined using a one‐way ANOVA followed by Tukey's post hoc test for multiple‐group comparisons. ^*^ *p* <0.05, ^**^ *p* <0.01, ^***^ *p* <0.001.

The heatmap for all DEGs identified from the PND vs. Con and PCN vs. PND revealed extensive transcriptional remodeling across the three groups (Figure S12A); among them, the transcriptional activity of inflammation‐ and immune‐related cytokines showed marked upregulation following surgery, which was partially reversed after PCN treatment (Figure 5E). Functional enrichment analyses were subsequently performed to characterize the biological significance of these transcriptional changes. In the PND vs. control comparison, DEGs were mainly associated with cytokine and chemokine responses, leukocyte/neutrophil recruitment, inflammatory signaling, and cell death‐related processes, indicating immune‐inflammatory activation in the aged hippocampus after surgery (Figure S12B, C). In contrast, Gene Ontology (GO) analysis of PCN‐responsive DEGs revealed that downregulated genes were predominantly enriched in immune‐inflammatory and cell death‐related processes (Figure 5G), whereas upregulated genes were associated with regulatory and metabolic functions (Figure 5F). Kyoto Encyclopedia of Genes and Genomes (KEGG) enrichment analysis of the downregulated DEGs further demonstrated that PCN modulated multiple inflammation‐, immune‐, and cell death‐associated pathways, including TNF, JAK‐STAT, Toll‐like receptor, NOD‐like receptor, and IL‐17 signaling pathways (Figure 5I), while enrichment analysis of upregulated DEGs revealed that PCN activates the PI3K/Akt signaling pathway (Figure 5H). Gene Set Enrichment Analysis (GSEA) further supported the transcriptomic findings by demonstrating altered pathway‐level gene signatures after PCN treatment. Compared with PND mice, PCN treatment reduced the enrichment of apoptosis‐, necroptosis‐, and innate immune‐related gene sets (Figure S12D–J), suggesting that PCN alleviates PND by suppressing coordinated inflammatory and cell death‐associated transcriptional responses.

Among the enriched inflammation‐ and immune‐related signaling pathways, the JAK2/STAT3 signaling cascade was further validated at the protein level, given its reported involvement in postoperative neuroinflammation [41, 42]. Western blot analysis showed that PCN treatment markedly reduced the phosphorylation levels of JAK2 and STAT3, indicating attenuation of surgery‐induced JAK2/STAT3 signaling activation (Figure 5J). In addition, PCN decreased AKT phosphorylation, further supporting the suppression of inflammation‐associated signaling cascades (Figure S12K,L). Collectively, these findings demonstrate that PCN alleviates PND by remodeling immune‐inflammatory transcriptional networks, suppressing pathological cell death programs, and attenuating key inflammatory signaling pathways.

### 2.5 PCN Regulates Microglial Glucometabolic Reprogramming to Limit Pro‐Inflammatory Polarization

Building on the pronounced therapeutic efficacy of PCN in ameliorating cognitive deficits and neuroinflammation in PND, together with the transcriptomic signatures implicating immune regulation, we next focused on microglia. Microglia exist in a highly dynamic surveillance state, continuously monitoring the brain microenvironment and actively participating in synaptic activity, pruning, and remodeling [43]. Consequently, aberrant microglial activation represents a critical contributor to CNS homeostatic imbalance and neuroinflammatory pathology [44]. Consistently, immunofluorescence analysis revealed a marked increase in pro‐inflammatory and phagocytic microglial populations in the aged hippocampus following PND induction (Figure 6A–D). Treatment with Pd/hCeO_2_ or PCN reduced microglia activation, with PCN exhibiting a superior suppressive effect (Figure 6A–D).

**FIGURE 6:**
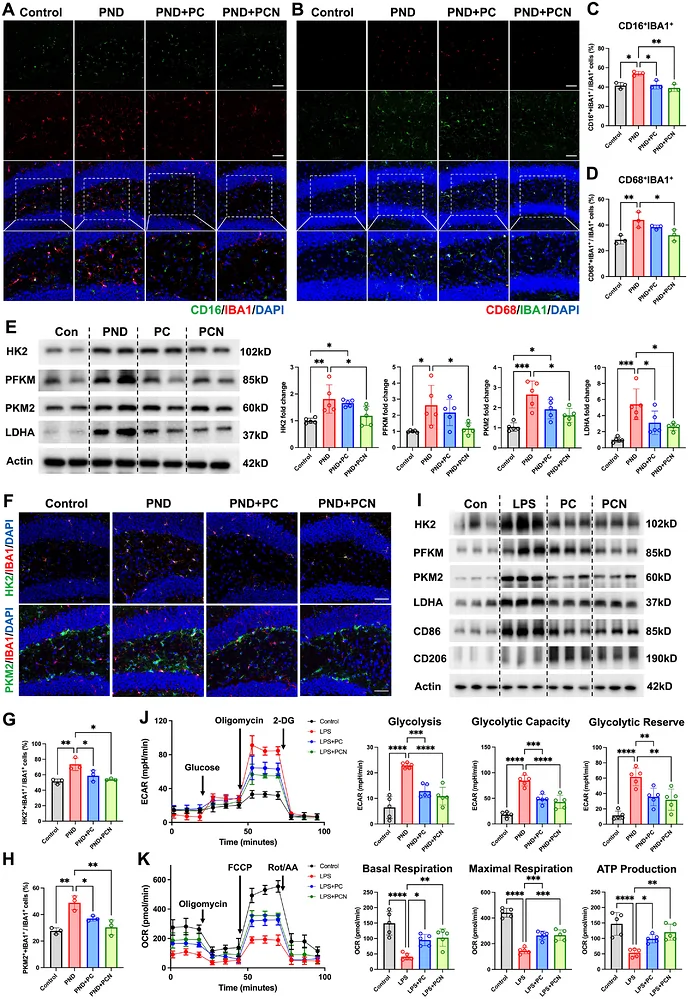
PCN attenuates microglial activation and regulates glucometabolic profiles. (A–D) Representative immunofluorescence images and quantification showing pro‐inflammatory and phagocytic microglia identified by CD16^+^/Iba1^+^ and CD68^+^/Iba1^+^ co‐staining in the dentate gyrus (DG) region of the hippocampus (*n* = 3). Scale bars: 50 µm. (E) Western blot analysis of glycolysis‐related enzymes (HK2, PFKM, PKM2, and LDHA) in hippocampal tissues from different groups (*n* = 5). (F–H) Representative immunofluorescence images and quantification showing HK2^+^/Iba1^+^ and PKM2^+^/Iba1^+^ microglia in the hippocampal DG region (*n* = 3). Scale bars: 50 µm. (I) Western blot analysis of glycolysis‐related enzymes (HK2, PFKM, PKM2, and LDHA) and microglial phenotype markers (CD86 and CD206) in BV2 cells following LPS stimulation with or without Pd/hCeO_2_ (PC) or PCN treatment (*n* = 3). (J, K) Seahorse extracellular flux analysis of BV2 cells after the indicated treatments, showing ECAR‐derived glycolytic parameters (glycolysis, glycolytic capacity, and glycolytic reserve) and OCR‐derived mitochondrial respiration parameters (basal respiration, maximal respiration, and ATP production) (*n* = 5). Data are presented as means ± SD. Statistical significance was analyzed by two‐way ANOVA followed by Tukey's multiple comparisons test. ^*^ *p* <0.05, ^**^ *p* <0.01, ^***^ *p* <0.001, and ^****^ *p* <0.0001.

Because microglial inflammatory phenotypes are tightly coupled to their metabolic state, and glycolysis‐driven metabolic reprogramming has been shown to mediate hippocampal microglial pro‐inflammatory polarization following surgical trauma in PND [45], we next examined whether PCN could modulate glycolysis‐associated microglial reprogramming following surgery. In the hippocampus of aged PND mice, expression of key glycolytic enzymes, including hexokinase 2 (HK2), phosphofructokinase muscle type (PFKM), pyruvate kinase M2 (PKM2), and lactate dehydrogenase A (LDHA), was markedly elevated (Figure 6E–H), indicating enhanced glycolytic activity associated with postoperative neuroinflammation. PCN administration significantly suppressed the upregulation of these glycolytic enzymes, suggesting effective attenuation of glycolysis‐driven microglial activation (Figure 6E–H). Consistent results were obtained in vitro using BV2 microglial cells. LPS stimulation induced a pro‐inflammatory phenotype, as evidenced by increased expression of the surface marker CD86 and upregulation of glycolytic enzymes (Figure 6I and Figure S13). PCN treatment significantly reduced glycolytic enzyme expression, accompanied by downregulation of CD86 and upregulation of the anti‐inflammatory marker CD206 (Figure 6I and Figure S13), indicating a phenotypic shift from a pro‐inflammatory to an anti‐inflammatory state.

Seahorse extracellular flux assays were conducted in the BV2 cultures to further characterize glucometabolic reprogramming. Following Pd/hCeO_2_ or PCN treatment, the extracellular acidification rates (glycolysis rate, glycolytic capacity, and glycolytic reserve) were significantly decreased, whereas the oxygen consumption rates (basal respiration, maximal respiration, and ATP production) were concomitantly elevated (Figure 6J, K), indicating that PCN effectively restored mitochondrial oxidative metabolism. Besides, in the in vitro model of microglia, PCN and Pd/hCeO_2_ exhibited nearly equivalent therapeutic effects. In aggregate, these findings demonstrate that inflammatory stimulation drives microglial polarization toward a pro‐inflammatory, glycolysis‐dependent state, whereas PCN effectively reverses this metabolic and functional reprogramming. This targeted modulation of microglial immunometabolism underscores the potent anti‐inflammatory efficacy of PCN and highlights its mechanistic basis for mitigating neuroinflammation and cognitive impairment in aged PND.

### 2.6 PCN Preserves BBB Integrity and Exhibits Excellent Biocompatibility

Activated microglia compromise BBB integrity by impairing endothelial function and promoting peripheral inflammatory infiltration, forming a self‐amplifying loop between neuroinflammation and barrier disruption in PND [46, 47]. Given the critical role of BBB disruption in amplifying postoperative neuroinflammation and cognitive decline, we next examined whether PCN treatment could mitigate surgery‐induced BBB impairment in aged PND mice. Immunofluorescence staining and western blot analyses revealed that PND induction resulted in a marked loss of the tight junction proteins, including zonula occludens‐1 (ZO‐1) and occludin (Figure 7A–C). In contrast, PCN administration significantly restored the expression of these junctional proteins in hippocampal microvessels, whereas Pd/hCeO_2_ alone exerted a weaker protective effect (Figure 7A–C). These findings indicate that PCN effectively preserves BBB structural integrity following surgical trauma.

**FIGURE 7:**
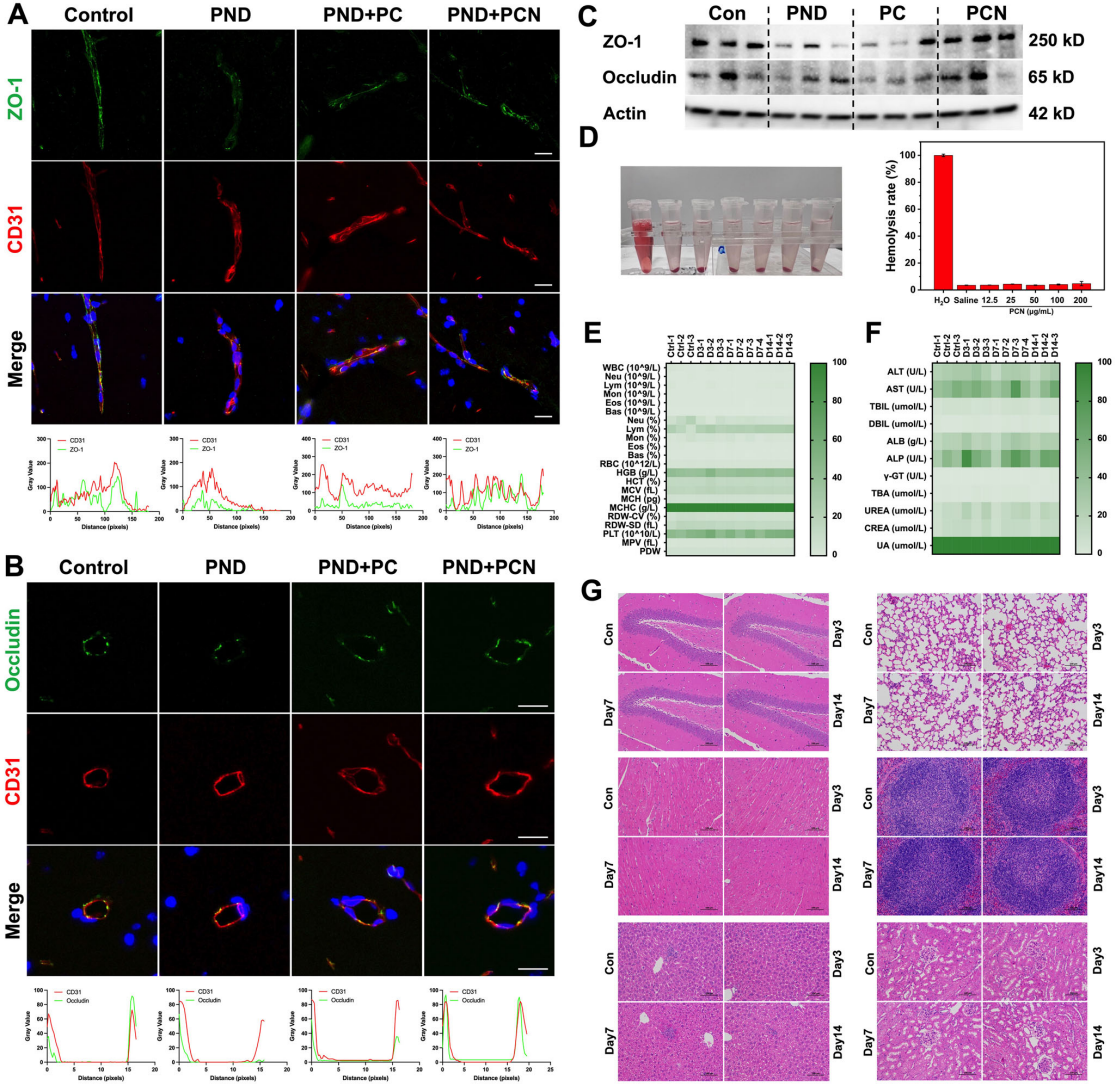
PCN preserves BBB integrity and exhibits excellent biocompatibility. (A, B) Representative immunofluorescence images showing the colocalization of the tight junction proteins ZO‐1 and occludin with vascular endothelial cells (CD31^+^) in the hippocampus following PND induction and treatment with Pd/hCeO_2_ (PC) or PCN. Scale bars, 20 µm. (C) Western blot analysis of tight junction proteins ZO‐1 and occludin expression in hippocampal tissues from the indicated groups. (D) Hemolysis assay evaluating the hemocompatibility of PCN at the indicated concentrations. (E, F) Routine hematological parameters and serum biochemical indices, including liver and kidney function markers, measured at 3, 7, and 14 days after PCN administration. (G) Representative H&E staining of major organs (brain, heart, liver, lung, spleen, and kidney) showing tissue morphology after PCN treatment. Data are presented as means ± SD (*n* = 3).

Beyond therapeutic efficacy, biosafety is a critical prerequisite for translational nanomedicine. Hemolysis assays demonstrated that PCN induced negligible erythrocyte lysis across a wide concentration range, indicating excellent hemocompatibility (Figure 7D). Consistently, routine blood examinations revealed no significant alterations in leukocyte, erythrocyte, or platelet counts at 3, 7, and 14 days after PCN administration (Figure 7E). Serum biochemical analyses further showed that key indicators of hepatic and renal function, including alanine aminotransferase, aspartate aminotransferase, blood urea nitrogen, and creatinine, remained within normal ranges in PCN‐treated mice (Figure 7F). Histopathological examination of major organs, including brain, heart, liver, spleen, lung, and kidney, revealed no detectable structural abnormalities or inflammatory infiltration after PCN treatment, confirming the absence of overt tissue toxicity (Figure 7G). Together, these results demonstrate that PCN possesses a favorable systemic safety profile. By integrating inflammation‐targeted delivery, neurovascular protection, and low immunotoxicity, PCN represents a promising and translatable nanotherapeutic strategy for PND in the aging population.

## 3 Conclusions

In summary, this study establishes a biomimetic nanozyme strategy that effectively counteracts aged PND by targeting the interconnected immunometabolic and neurovascular mechanisms underlying postoperative neuroinflammation. By cloaking Pd/hCeO_2_ nanozyme cores with homologous, highly adhesive neutrophil membranes, PCN achieves inflammation‐guided brain delivery, enabling precise intervention at sites of postoperative neurovascular injury. Mechanistically, PCN suppresses excessive ROS and attenuates glycolysis‐driven pro‐inflammatory microglial activation by reprogramming microglial immunometabolism toward a more homeostatic state. This metabolic modulation disrupts the pathogenic feed‐forward loop linking microglial activation, blood–brain barrier breakdown, and peripheral inflammatory infiltration, thereby preserving synaptic structure, neuronal viability, and BBB integrity. At the functional level, these coordinated effects translate into robust improvements in learning and memory in aged PND mice. PCN exhibits favorable biosafety and systemic tolerability, underscoring its translational promise as a biosafe and low‐immunogenic nanotherapeutic platform. Collectively, our findings not only provide mechanistic insight into the immunometabolic basis of PND but also highlight inflammation‐targeted nanozyme therapy as a viable and broadly applicable approach for preventing surgery‐induced cognitive decline in the aging brain.

Nevertheless, several limitations should be acknowledged. First, although the therapeutic efficacy of PCN was demonstrated in aged murine PND models, further validation in larger animal models and clinically relevant surgical paradigms is required. Second, while microglial immunometabolic modulation was identified as a key mechanism, the involvement of other neurovascular unit components and upstream regulatory pathways remains to be elucidated. Finally, from a translational perspective, the long‐term biodistribution, biodegradability, and safety of PCN warrant comprehensive investigation to facilitate clinical application.

## 4 Materials and Methods

Other detailed information and methods are available in the Supplementary Information.

### 4.1 Synthesis of Pd/hCeO_2_ Nanospheres

Hollow cerium oxide (hCeO_2_) nanospheres were fabricated via a modified solvothermal strategy based on a previously reported protocol. In brief, cerium nitrate hexahydrate (1 g) and polyvinylpyrrolidone (PVP, 0.4 g) were dissolved in a mixed solvent consisting of 30 mL ethylene glycol and 2 mL of 1 m hydrochloric acid under continuous stirring to form a homogeneous solution. The resulting mixture was transferred into a Teflon‐lined stainless‐steel autoclave and maintained at 160°C for 3 h. After naturally cooling to room temperature, the precipitates were collected by centrifugation, washed repeatedly with acetone and deionized water, and subsequently freeze‐dried to obtain hCeO_2_ nanoparticles.

For the preparation of Pd‐loaded hCeO_2_ nanospheres, 40 mg of the as‐prepared hCeO_2_ was dispersed in 8 mL of deionized water containing Na_2_PdCl_4_ (20 mg), PVP (20 mg), ascorbic acid (20 mg), and KCl (65 mg). The suspension was subjected to ultrasonic treatment to ensure uniform dispersion, followed by heating at 80°C for 3 h to facilitate in situ reduction and deposition of Pd nanoparticles. The resulting Pd/hCeO_2_ nanospheres were collected by centrifugation, thoroughly washed with deionized water, and dried for subsequent characterization and biological experiments.

### 4.2 Synthesis of PCN Nanoformulation

Neutrophil membranes were isolated from freshly obtained mouse neutrophils using differential centrifugation. Briefly, neutrophils were isolated from 8‐week‐old C57BL/6 mice following LPS stimulation to enhance neutrophil activation. 6 h after intraperitoneal injection of LPS, femurs and tibias were harvested, and bone marrow cells were flushed out using HBSS containing 0.38% sodium citrate. After filtration through a 70 µm mesh, cells were layered onto a discontinuous Percoll gradient (52%, 64%, and 72%) and centrifuged to isolate neutrophils. Neutrophils were collected from the 64%–72% interface, washed, and treated with red blood cell lysis buffer. The purified neutrophils were subjected to repeated freeze‐thaw cycles to disrupt the cell membrane, followed by centrifugation to remove intracellular components. The collected membrane fraction was washed with cold PBS and resuspended for subsequent use.

The isolated neutrophil membranes were then mixed with Pd/hCeO_2_ nanoparticles at a mass ratio of 1:5 and extruded successively through polycarbonate membranes with pore sizes of 800, 400, and 200 nm using a mini extruder to achieve uniform membrane coating. The resulting neutrophil membrane‐camouflaged nanoparticles (PCN) were collected and purified to remove free membranes and aggregates. Successful fabrication of PCN was confirmed by morphological characterization and protein analysis. The presence of neutrophil‐specific membrane proteins on the surface of PCN, including β2‐integrin, CD11a, CD11b, and CD44, was verified by western blotting.

### 4.3 Animals and Treatment

Animal experiments in the present study were approved by the Institutional Animal Care and Use Committee of Renji Hospital (RJ2026‐035), Shanghai Jiao Tong University School of Medicine. All animal experiments were conducted in compliance with animal care guidelines. Male C57BL/6 mice (18 months old, 30–35 g; Bikai‐keyi Biotechnology Co., Ltd., Shanghai, China) were housed in standard cages under controlled laboratory conditions (temperature of 22 ± 2°C, 12‐h light/12‐h dark cycle) with free access to regular rodent pellets and water. All mice were allowed to adapt to their new environment for 7 days before the experiments were initiated.

Mice in the surgery group were subjected to tibial fracture and fixation under isoflurane anesthesia to establish the PND model as previously described [48]. Briefly, mice were anesthetized with 2.1% isoflurane in 30% oxygen and received subcutaneous butorphanol (0.1 mg/kg) for analgesia prior to surgery. A longitudinal incision was made on the left hind limb, and the tibia was exposed by blunt muscle dissection. A 0.38‐mm stainless steel pin was inserted into the intramedullary canal, followed by a standardized tibial osteotomy. The incision was irrigated and closed with 6‐0 sutures. Mice were maintained on a warming pad until recovery. To achieve targeted intervention, Pd/hCeO_2_ or PCN (1 mg/kg) was intravenously administered via the tail vein at 2 h and 24 h after surgery.

### 4.4 Statistical Analysis

All data are expressed as mean ± standard deviation (SD), except for Morris Water Maze escape latency and speed, which are presented as mean ± standard error of the mean (SEM). The sample size and statistical analysis for each experiment were indicated in the figures and figure legends. Normality of datasets was assessed using the Shapiro‐Wilk test. For comparisons between two groups, an unpaired two‐tailed Student's *t*‐test was applied. For multiple group comparisons, one‐way or two‐way analysis of variance (ANOVA) followed by Tukey's post hoc test was used as appropriate. Two‐way ANOVA with repeated measures was used to analyze the water maze escape latency and speed. All statistical analyses were performed using GraphPad Prism 10.0 (GraphPad Software, USA), and differences were considered statistically significant at *p* < 0.05.

## Author Contributions

**L.H., J.C., and H.X**. contributed equally. **B.L., Q.Z., D.S**., and **D.W**. designed the project. **L.H., J.C., and H.X**. performed most of the experiments. **Q.Z. and Z.X**. synthesized and characterized the materials. **X.Z. and Y.L**. conducted RNA sequencing and bioinformatics analyses. **Y.Z., W.D., Z.X., L.G., L.X., W.W., and X.X**. performed data collection and analysis. **L.H. and B.L**. drafted the manuscript. **W.Y. and Y.Z**. assisted in supervising the experiments. **B.L., D.S., and D.W**. supervised the study and revised the manuscript. All authors discussed the results and commented on the manuscript.

## Conflicts of Interest

The authors declare no conflicts of interest.

## Supporting information




**Supporting File**: advs78033‐sup‐0001‐SuppMat.docx.

## Data Availability

Figure 1 and ToC were created in BioRender. Huang, L. (2026) https://BioRender.com/u8g59kf; Figure 3A was created in BioRender. Huang, L. (2026) https://BioRender.com/1147dq7. The RNA‐sequencing data generated in this study have been deposited in the NCBI Gene Expression Omnibus (GEO) database under accession number GSE339068. Other data supporting the findings of this study are available from the corresponding author upon reasonable request.
