# Supplementary material for: A Neutrophil Membrane‐Encapsulated Pd/hCeO2 Nanoformulation for Alleviating Age‐Dependent Perioperative Neurocognitive Disorders
Source: Adv Sci (Weinh). 2026 Sep 30:e78033. Online ahead of print. doi: 10.1002/advs.78033 (PMC13627777; doi:10.1002/advs.78033)
Supplement: Supplementary file 1 [file ADVS-9999-e78033-s001.docx]

**Supporting Information**

**A Neutrophil Membrane-Encapsulated Pd/hCeO_2_ Nanoformulation for Alleviating Age-Dependent Perioperative Neurocognitive Disorders**

Lili Huang^1, 2, #^, Jiahui Cheng^3, #^, Huan Xu^4, #^, Qiang Zhang^5, *^, Xiao Zhang^6^, Ye Liu^6^, Yizhe Zhang^1, 2^, Wanbing Dai^1, 2^, Zhi Xiong^5^, Linghuan Guo^5^, Lingling Xu^3^, Wen Wang^3^, Xin Xu^3^, Yan Zhou^3, 7^, Weifeng Yu^1, 2^, Bo Li^2, 3, *^, Diansan Su^6, *^, Daxiang Wen^1, 2, *^

1. Department of Anesthesiology, Renji Hospital, Shanghai Jiao Tong University School of Medicine; No. 160, Pujian Road, Pudong District, Shanghai 200127, China
2. Key Laboratory of Anesthesiology (Shanghai Jiao Tong University), Ministry of Education; No. 160, Pujian Road, Pudong District, Shanghai 200127, China
3. Department of Radiology, Renji Hospital, Shanghai Jiao Tong University School of Medicine; No. 160, Pujian Road, Pudong District, Shanghai 200127, China
4. Department of Anesthesiology, Shanghai Pulmonary Hospital, Tongji University School of Medicine; No. 507, Zhengmin Road, Yangpu District, Shanghai 200433, China
5. Institute of Diagnostic and Interventional Radiology, Shanghai Sixth People's Hospital, Shanghai Jiao Tong University School of Medicine; No. 600, Yishan Road, Xuhui District, Shanghai 200233, China
6. Department of Anesthesiology, The First Affiliated Hospital, Zhejiang University School of Medicine, No.79, Qingchun Road, Hangzhou 310003, China
7. College of Health Science and Technology, Shanghai Jiao Tong University School of Medicine; No. 227, Chongqingnan Road, Huangpu District, Shanghai 200025, China

#. These authors contributed equally: Lili Huang, Jiahui Cheng, and Huan Xu

**Corresponding Author:** Qiang Zhang, zhangqiang@shanghaitech.edu.cn; Bo Li, 1993818@sjtu.edu.cn; Diansan Su, diansansu@yahoo.com; Daxiang Wen, wdxrwj@126.com

**This PDF file includes:**

Supplementary materials and methods

Supplementary Figures 1 to 13

**Supplementary materials and methods**

**Characterizations**

The morphology and structural features of hCeO_2_, Pd/hCeO_2_, and PCN were examined using a high-resolution transmission electron microscopy (HRTEM, Tecnai G2). Elemental distribution and compositional analysis were further conducted by scanning transmission electron microscopy equipped with energy-dispersive X-ray spectroscopy (STEM-EDS, JEM-3200FS, JEOL, Japan). The hydrodynamic size distribution of nanoparticles was determined by dynamic light scattering (DLS) using a SZ-100 nanoparticle analyzer (HORIBA Scientific). Crystalline structures of Pd/hCeO_2_ were analyzed by X-ray diffraction (XRD) using a Rigaku D/max 2500 diffractometer (Bruker, Germany) with Cu Kα radiation (λ = 1.5418 Å) operated at 40 kV and 40 mA. Surface chemical composition and valence states were characterized by X-ray photoelectron spectroscopy (XPS) performed on a Thermo Scientific K-Alpha system equipped with an Al Kα X-ray source (1486.6 eV). Binding energies were calibrated using the C 1s peak at 284.8 eV as an internal reference. Ultraviolet-visible (UV-Vis) absorption spectra were recorded using a Shimadzu UV-2501 spectrophotometer to evaluate the optical properties of the nanomaterials.

**In vivo biodistribution and imaging of PCN**

The in vivo biodistribution of PCN was evaluated using fluorescence imaging. Briefly, aged C57BL/6 mice (18 months old) were randomly divided into three groups, control, control+PCN and PND+PCN groups. Cy5.5-labeled PCN (1 mg/kg) was intravenously administered via the tail vein at 2 h after tibia fracture surgery. At 12 h and 24 h post-injection, mice were anesthetized and subjected to in vivo fluorescence imaging using an IVIS imaging system (Vieworks, Korea) to assess the systemic distribution of PCN. Immediately after imaging, the animals were euthanized, and major organs including the heart, liver, spleen, lungs, kidneys, and brain were harvested for ex vivo fluorescence imaging. The fluorescence intensity of each organ was quantitatively analyzed to evaluate the biodistribution profile and tissue accumulation of PCN. Special attention was given to cerebral fluorescence signals to determine the brain-targeting capability of PCN under postoperative inflammatory conditions. All imaging data were processed using the manufacturer’s software, and fluorescence intensities were normalized for comparison among different groups.

**ROS scavenging assays**

The reactive oxygen species (ROS) scavenging performance of Pd/hCeO_2_ and PCN was evaluated by electron spin resonance (ESR) spectroscopy using a Bruker ESR 5000 spectrometer. The ability of PCN to eliminate hydroxyl radicals (•OH) and superoxide anions (•O₂⁻) was examined with 5-dimethyl-1-pyrroline-N-oxide (DMPO) serving as the spin-trapping reagent. For hydroxyl radical detection, DMPO (5 μL, 10 M) was mixed with PBS (45 μL, 10 mM). Hydroxyl radicals were generated through a Fenton-like reaction by sequential addition of Fe²⁺ solution (20 μL, 50 mM) and H₂O₂ (30 μL, 50 mM). ESR spectra were recorded in the presence or absence of Pd/hCeO_2_ or PCN, and the scavenging efficiency was evaluated based on the attenuation of characteristic DMPO-•OH signals. •O₂⁻ were produced by dissolving potassium superoxide (70 μg) in 200 μL of 18-crown-6/DMSO solution (0.7 mM). After the addition of DMPO (50 μL), ESR measurements were performed to assess the SOD-like activity of Pd/hCeO_2_ and PCN by comparing signal intensities with and without nanoparticle treatment. In addition, the CAT-like activity of PCN was evaluated using a hydrogen peroxide detection kit (Solarbio, Beijing). Briefly, residual H_2_O_2_ reacted with titanium sulfate to form a yellow titanium-peroxide complex, whose absorbance was measured at 415 nm using a UV-vis spectrophotometer. Lower absorbance values indicated enhanced decomposition of H_2_O_2_, reflecting stronger CAT-mimetic activity of PCN.

**Behavioral assessment**

Cognitive function and exploratory behavior were evaluated using a battery of behavioral tests, including the Morris water maze (MWM), novel object recognition (NOR), and open field test (OFT). All behavioral experiments were conducted in a sound-attenuated room under controlled lighting conditions, and animals were allowed to acclimate to the testing environment prior to assessment. Behavioral data were recorded and analyzed using the AnyMaze tracking system (Stoelting Co., USA).

**Open field test (OFT)**

Spontaneous locomotor activity and anxiety-related behavior were assessed using an open field paradigm. Mice were individually introduced into the center of a square arena (45 × 45 × 45 cm³) and allowed to explore freely for 10 min. The central region, defined as 50% of the total arena area, was used for anxiety-related analysis. Locomotor trajectories were continuously tracked, and total travel distance together with center-zone occupancy time were quantified. The arena was cleaned thoroughly between trials to prevent olfactory interference.

**Morris water maze test (MWM)**

The MWM test was performed to evaluate spatial learning and memory as previously described with minor modifications.^[1]^ The apparatus consisted of a circular pool (120 cm in diameter) filled with water to a depth of 40 cm, maintained at 23 ± 1°C. The water was rendered opaque using non-toxic white milk powder. The pool was in a testing room containing stable visual cues and was virtually divided into four equal quadrants.

A circular escape platform (8 cm in diameter) was submerged 1 cm below the water surface and positioned in the center of one designated quadrant. During the training phase, mice underwent four training trials per day for four consecutive days. In each trial, mice were allowed a maximum of 60 s to locate the hidden platform. If unsuccessful, they were gently guided to the platform and allowed to remain there for 15 s. The inter-trial interval was 20 min. On day 5, a probe test was conducted in which the platform was removed. Mice were allowed to swim freely for 60 s to assess memory retention. Parameters including swimming speed, latency to reach the platform location, number of platform zone crossings, and time spent in each quadrant were automatically recorded and analyzed.

**Novel object recognition test (NOR)**

Recognition memory was evaluated using the NOR test in a square arena (45 × 45 × 45cm³). On day 1, mice were habituated to the empty arena. On day 2, two identical objects were placed in opposite corners, and mice were allowed to explore for 10 min. After a retention interval of 1 h, one familiar object was replaced with a novel object, and mice were reintroduced for a 10 min test session. Object exploration was defined as nose-oriented investigation within 1 cm of the object, excluding climbing or sitting behaviors. All sessions were video-recorded and analyzed in a blinded manner. Recognition memory was quantified using the discrimination index (DI) = (Tn − Tf) / (Tn + Tf) × 100%. Animals with total exploration times below 10 sec were excluded from analysis.

**Enzyme-linked immunosorbent assay**

At 48 h postoperatively, aged mice were deeply anesthetized, and blood was collected by cardiac puncture into EDTA-coated tubes. Plasma was obtained by centrifugation (3,000 ×g, 15 min, 4°C) and stored at -80°C. Mice were then transcardially perfused with cold PBS, and brains were rapidly harvested. Hippocampal tissues were dissected on ice, homogenized in lysis buffer containing protease inhibitors, and centrifuged (12,000 ×g, 15 min, 4°C). Supernatants were collected, and total protein concentrations were determined using a BCA assay. Levels of interleukin-1β (IL-1β), IL-6, and tumor necrosis factor-α (TNF-α) in plasma and brain lysates were quantified using commercially available ELISA kits (Multi science, Cat# EK201B, EK206, EK282, China) according to the manufacturers’ instructions. Absorbance of each sample was measured at 450 nm, and the cytokine levels in brain tissues were normalized to total protein content.

**Western blot analysis**

Brain tissues or cells were lysed in ice-cold RIPA buffer containing protease and phosphatase inhibitors. Lysates were centrifuged at 12,000 ×g for 15 min at 4°C, and protein concentrations were determined by a bicinchoninic acid protein assay kit (Thermo Fisher Scientific, United States). Equal amounts of protein were separated by SDS–PAGE and transferred onto PVDF membranes (pore size: 0.22 µm; Millipore, United States), which were blocked with 5% milk or BSA in TBST and incubated overnight at 4°C with primary antibodies against PSD95 (1:1000, Abcam, ab18258, UK), SYN-1 (1:1000, ABclonal, A24122, China), SYP (1:1000, ABclonal, A19122, China), HK2 (1:1000, Abcam, ab209847, UK), PKM2 (1:1000, CST, 4053S, United States), PFKM (1:1000, ABclonal, A5477, China), LDHA (1:1000, ABclonal, A1146, China), ZO-1 (1:1000, Abcam, ab96587, UK), Occludin (1:1000, Abcam, ab216327, UK), p-AKT (1:1000, CST, 4060S, United States), total AKT (1:1000, CST, 9272S, United States), p-JAK2 (1:500, ABclonal, AP0531, China), total JAK2 (1:1000, ABclonal, A7694SP, China), p-STAT3 (1:500, ABclonal, AP0705, China ), STAT3 (1:1000, Proteintech, 10253-2-AP, China), CD44 (1:1000, ABclonal, A12410, China), CD11a (1:1000, ABclonal, A23960, China), CD11b (1:1000, CST, 93169S, United States), β2-integrin (1:1000, CST, 72607S, United States), ATP1A1 (1:2000, Genuin Biotech, 71282, China), VCAM1 (1:1000, Abcam, ab134047, UK), ICAM1 (1:5000, ABclonal, A26412PM, China), CD86 (1:1000, ABclonal, A2353, China), CD206 (1:1000, CST, 24595S, United States), and β-actin-HRP (1:50000, Abcam, ab49900, UK). After washing, membranes were incubated with HRP-conjugated secondary antibodies for 1 h at room temperature. Bands were visualized by enhanced chemiluminescence and ChemiDoc XRS system (Bio-Rad, United States), the band intensities were quantified using ImageJ, with phosphorylated proteins normalized to total protein and other targets normalized to loading controls.

**Immunohistochemistry**

Mice were anesthetized and transcardially perfused with cold PBS followed by 4% paraformaldehyde (PFA). Brains were post-fixed overnight, cryoprotected in 30% sucrose, and coronally sectioned at 30 µm through the hippocampus. Sections were permeabilized with 0.3% Triton X-100, blocked with 5% BSA, and incubated overnight at 4°C with primary antibodies against PSD95 (1:500, Abcam, ab18258, UK), β3-Tubulin (1:500, Abcam, ab78078, UK) , IBA1 (1:500, Abcam, Ab5076, UK), CD16 (1:200, Santa Cruz, sc-58962 , United States), CD68 (1:100, Abcam, Ab53444, UK), HK2 (1:500, Abcam, ab209847, UK), PKM2 (1:500, CST, 4053S, United States), ZO-1 (1:500, Abcam, ab96587, UK), Occludin (1:500, Abcam, ab216327, UK), or CD31 (1:800, RD systems, AF3628, United States). Sections were then incubated with species-matched fluorescent secondary antibodies, counterstained with DAPI, mounted, and imaged using a confocal microscope (Olympus FV3000, Japan). Fluorescence intensity and colocalization were quantified using ImageJ by investigators blinded to group allocation.

**Dihydroethidium (DHE) staining for hippocampal ROS detection**

DHE staining was performed to assess hippocampal ROS accumulation. Fresh hippocampal tissues were embedded in OCT compound, cryo-sectioned, and incubated with DHE staining solution (Beyotime Biotechnology, S0064S, China) at 37 °C for 30 min in the dark. After washing with PBS, fluorescence images were acquired under identical imaging conditions, and DHE fluorescence intensity was used to evaluate ROS levels.

**Terminal deoxynucleotidyl transferase-mediated dUTP nick-end labeling (****TUNEL) assay**

Apoptotic cells in the hippocampus were detected using a TUNEL assay kit (Servicebio, G1504-50T, China). Brain perfusion, fixation, cryoprotection, and sectioning were performed as described above for immunofluorescence staining. Briefly, free-floating sections were permeabilized and incubated with TUNEL reaction mixture at 37°C for 60 min in a humidified dark chamber, followed by DAPI counterstaining. DNase I-treated sections served as positive controls. Confocal images of hippocampal regions were obtained, and TUNEL-positive cells were quantified using ImageJ.

**Mitochondrial membrane potential assay**

Mitochondrial membrane potential (ΔΨm) was assessed using a JC-1 Mitochondrial Membrane Potential Assay Kit (MedChemExpress, HY-K0601, China). Briefly, HT22 cells were seeded in 6-well plates and treated as indicated. For positive control, cells were incubated with carbonyl cyanide 3-chlorophenylhydrazone (CCCP, 50 μM) for 5min at 37°C to induce mitochondrial depolarization. Subsequently, cells were incubated with JC-1 working solution (final concentration 2 μM) at 37°C for 15-20 min in the dark. After washing twice with PBS, cells were immediately analyzed by confocal microscopy or flow cytometry. Healthy mitochondria exhibited red fluorescence (JC-1 aggregates; Ex/Em=585/590 nm), whereas depolarized mitochondria displayed green fluorescence (JC-1 monomers; Ex/Em=510/527 nm). Mitochondrial membrane potential was evaluated based on the red/green fluorescence ratio.

**Transmission electron microscopy of hippocampal synapses**

For ultrastructural analysis of synapses, mice were deeply anesthetized and transcardially perfused with PBS followed by 2.5% glutaraldehyde. Hippocampal tissues were rapidly dissected and cut into about 1 mm³ blocks, then fixed in 2.5% glutaraldehyde at 4°C overnight. After rinsing, samples were post-fixed in 1% osmium tetroxide, dehydrated through a graded ethanol series, and embedded in epoxy resin. Ultrathin sections (~70nm) were collected on copper grids and counterstained with uranyl acetate and lead citrate. Synaptic ultrastructure was examined using a transmission electron microscope (TECNAI G2, FEI) operated at 200 kV. Synaptic parameters, including synaptic density, postsynaptic density thickness, and synaptic cleft width, were quantified from randomly selected fields using Image-Pro Plus 6.0 software.

**RNA sequencing analysis**

Hippocampal tissues from control, PND, and PND+PCN mice were snap-frozen in liquid nitrogen. Total RNA was extracted using the Universal RNA Extraction CZ Kit (RNU441, ONREW). RNA quality was assessed using a Qubit 4.0 Fluorometer (Invitrogen), and RNA quality was evaluated by electrophoresis on a denaturing agarose gel. Libraries were prepared using standard mRNA kits and sequenced on an Illumina NovaSeq platform to generate paired-end reads. Reads were quality-filtered, aligned to the mouse reference genome, and quantified as FPKM. Differentially expressed genes (DEGs) were identified using |log_2_ fold change| ≥ 1 and *P* ≤ 0.05. Functional enrichment analyses, including GO and KEGG, were performed to explore biological processes and pathways altered by PCN. Gene set enrichment analysis (GSEA) was used to assess coordinated changes in inflammation- and metabolism-related pathways.

**In vivo biocompatibility analysis**

To evaluate the in vivo biocompatibility of PCN, healthy aged mice were randomly assigned to a control group or PCN-treated groups and sacrificed at 3, 7, and 14 days after a single intravenous administration of PCN (10 mg/kg). At the designated time points, mice were anesthetized, and blood samples were collected for hematological and serum biochemical analyses, including routine blood parameters and liver and kidney function indicators. Subsequently, mice were perfused with PBS, and the brain, heart, liver, spleen, lung, and kidney were harvested, fixed in 4% paraformaldehyde, embedded in paraffin, sectioned, and stained with hematoxylin and eosin (H&E) to assess tissue morphology and potential histopathological alterations. In addition, the hemocompatibility of PCN was evaluated using a hemolysis assay. Fresh whole blood collected from healthy aged mice was incubated with PCN at different concentrations (0-200 ug/ml). After incubation, samples were centrifuged, and the absorbance of the supernatant was measured to calculate the hemolysis ratio, with deionized water and saline serving as positive and negative controls, respectively.

**Cell culture and treatment**

HT22 mouse hippocampal neuronal cells and BV2 mouse microglial cells were purchased from Procell Life Science & Technology Co., Ltd. (Wuhan, China) and maintained in DMEM supplemented with 10% fetal bovine serum and 1% penicillin–streptomycin at 37 °C with 5% CO_2_. To establish in vitro injury models, HT22 cells were exposed to H₂O₂ (200 μM) (Sigma-Aldrich, USA) to induce oxidative stress, whereas BV2 cells were stimulated with LPS (100 ng/mL) to induce inflammatory activation. Based on preliminary cytotoxicity evaluation, Pd/hCeO_2_ and PCN were applied at non-toxic concentrations for subsequent experiments.

**Cellular cytotoxicity, viability, and apoptosis assays**

HT22 cells were seeded in 96-well plates and treated with PC or PCN at concentrations of 0, 12.5, 25, 50, 100, and 200 μg/mL. Cell viability was evaluated using the Cell Counting Kit-8 (CCK-8, CK04, Dojindo, Japan). To establish an oxidative stress model, cells were exposed to H₂O₂ (200 μM; Sigma-Aldrich, USA) and subsequently subjected to Calcein-AM/PI live/dead staining (Beyotime Biotechnology, C2015, China). Apoptosis was quantified using an Annexin V-FITC/PI apoptosis detection kit (BD Biosciences, 556547, USA) according to the manufacturer’s instructions.

**Cellular uptake assay**

To evaluate the cellular uptake efficiency of PCN, FITC-labeled PCN (100 μg/mL) was incubated with HT22 neurons and BV2 microglia for different time periods (0, 1, 2, 4, and 8 h). After incubation, cells were detached using trypsin, collected, and washed with PBS to remove uninternalized nanoparticles. The intracellular FITC fluorescence was analyzed by flow cytometry (Agilent Technologies, Santa Clara, CA, USA), and the percentage of FITC-positive cells was quantified to assess the time-dependent cellular uptake of PCN.

**Intracellular ROS detection assay**

Intracellular ROS levels were detected using the ROS Assay Kit (DCFH-DA, Beyotime Biotechnology, S0033S, China). After treatment, cells were washed with PBS and incubated with DCFH-DA working solution at 37 °C in the dark according to the manufacturer’s instructions. After staining, cells were washed and subjected to flow cytometric analysis. For flow cytometry, cells were collected, resuspended in PBS, and analyzed using consistent gating strategies. The mean fluorescence intensity (MFI) of DCF was used to quantify intracellular ROS levels.

**Seahorse analysis**

BV2 cells (1 × 10⁴ cells/well) were seeded into a Seahorse XF96 microplate and allowed to adhere for 24 h. Cells were treated with control medium, LPS (100 ng/mL), LPS + PC (50 μg/mL), or LPS + PCN (50 μg/mL) for 4 h. After treatment, cells were centrifuged and the medium replaced with Seahorse XF assay medium (supplemented with 10 mM glucose, 1 mM pyruvate, and 2 mM L-glutamine). Real-time extracellular acidification rate (ECAR) and oxygen consumption rate (OCR) were measured using a Seahorse XF96 Analyzer (Agilent, USA) following the manufacturer’s instructions. Key parameters including basal glycolysis, glycolytic capacity, basal respiration, and maximal respiration were calculated.

**Supplementary figures**

**Figure S1. Quantitative analysis of the expression levels of neutrophil membrane-associated adhesion proteins.** NM, neutrophil membranes without inflammatory stimulation; INM, neutrophil membranes with inflammatory stimulation; PCN, Pd/hCeO_2_@highly adhesive neutrophil membrane. Data are presented as means **±** SD (*n* = 3 independent experiments). Statistical significance was determined by one-way ANOVA followed by Tukey’s post hoc test. **P* <0.05, ***P* <0.01, ****P* <0.001.

**Figure S2.** **Zeta potential of hCeO_2_, Pd/hCeO_2_, and PCN.**

**Figure S3.** ***Ex vivo* fluorescence imaging of major organs at 12 h after PCN administration.** Representative images and quantitative analysis of relative fluorescence intensity in major organs (From top to bottom: heart, liver, spleen, lung, kidney) of normal aged and PND mice at 12 h after PCN administration. Data are presented as means **±** SD (*n* = 3). Statistical significance was determined by one-way ANOVA followed by Tukey’s post hoc test. **P* <0.05, ***P* <0.01, ****P* <0.001, *****P* <0.0001.

**Figure S4. *Ex vivo* fluorescence imaging of major organs at 24 h after PCN administration.** Representative images and quantitative analysis of relative fluorescence intensity in major organs (From top to bottom: heart, liver, spleen, lung, kidney) of normal aged and PND mice at 24 h after PCN administration. Data are presented as means **±** SD (*n* = 3). Statistical significance was determined by one-way ANOVA followed by Tukey’s post hoc test. **P* <0.05, ***P* <0.01, ****P* <0.001, *****P* <0.0001.

**Figure S5. Time-dependent cellular uptake of PCN in HT22 neurons and BV2 microglia.** Representative flow cytometry histograms showing the uptake of FITC-labeled PCN by HT22 neurons and BV2 microglia after incubation for 0, 1, 2, 4, and 8 h (*n* = 3). The proportion of FITC-positive cells gradually increased with prolonged incubation, demonstrating efficient time-dependent cellular uptake of PCN in both neuronal and microglial cell models.

**Figure S6. Flow cytometric analysis of intracellular ROS levels in BV2 cells after different treatments.** Representative flow cytometry histograms of intracellular ROS levels in BV2 cells treated with hCeO_2_, Pd/hCeO_2_ (PC), or PCN. Intracellular ROS levels were detected by DCFH-DA staining (*n* = 3).

**Figure S7. Behavioral performance in the Morris water maze.** (A) Swimming speed during the training phase showed no significant differences among groups (*n* = 16). (B) Platform crossing numbers in the probe trial. PND mice displayed reduced crossings compared with controls, which were partially restored following PC or PCN treatment (*n* = 16). Data are presented as means **±** SEM for MWM training speed data, and means **±** SD for crossing times data. Speed data was analyzed using two-way repeated-measures ANOVA followed by Tukey’s multiple comparisons test, while platform crossings were analyzed using one-way ANOVA followed by Tukey’s multiple comparisons test. **P* <0.05, ***P* <0.01, ****P* <0.001, *****P* <0.0001.

**Figure S8. PCN attenuates surgery-induced oxidative stress in the hippocampus of aged PND mice.** Representative dihydroethidium (DHE) staining images of hippocampal sections from the indicated groups (*n* = 3). Scale bar: 50 μm.

**Figure S9. PC or PCN treatment attenuates oxidative stress-induced synaptic protein loss in vitro.** HT22 cells were exposed to H₂O₂ to induce oxidative stress, followed by treatment with PC or PCN. The expression levels of PSD95 and synaptophysin (SYP) were analyzed, showing a reduction after H₂O₂ exposure and marked recovery upon PC or PCN treatment. Data are presented as means **±** SD (*n* = 3 independent experiments). Statistical significance was determined by one-way ANOVA followed by Tukey’s post hoc test. **P* <0.05, ***P* <0.01, ****P* <0.001, *****P* <0.0001.

**Figure S10. Cytocompatibility and anti-apoptotic effects of PCN in HT22 cells.** (A) CCK-8 assay showing the viability of HT22 cells after incubation with increasing concentrations of PCN for 24 h (*n* = 6 independent experiments). (B) HT22 cells were pretreated with increasing concentrations of PCN (0-200μg/mL) and then exposed to 200μM H_2_O_2_. PCN dose-dependently increased cell viability (*n* = 6 independent experiments). (C) Flow cytometric analysis of apoptosis in HT22 cells exposed to H_2_O_2_ with or without PC or PCN treatment. (D) Calcein-AM/PI staining showing dose-dependent cytoprotective effects of PCN against H_2_O_2_-induced neuronal injury (*n* = 3 independent experiments). Scale bar: 20 μm. Data are presented as means **±** SD.

**Figure S11. PCN preserves** **mitochondrial membrane potential in HT22 cells under oxidative stress.** (A) Representative JC-1 fluorescence images of HT22 cells under indicated treatments (*n* = 3). Scale bar: 20 μm. (B) Representative flow cytometry profiles of JC-1 staining in HT22 cells (*n* = 3). H_2_O_2_ treatment shifted fluorescence toward the green channel, indicating mitochondrial membrane potential loss, while PCN treatment partially restored the red fluorescence signal. Data are presented as means **±** SD.

**Figure S12. Transcriptomic analysis reveals PCN-mediated modulation of inflammation- and immune-related alterations in PND.** (A) Heatmap showing the expression patterns of all differentially expressed genes (DEGs) identified from the PND vs. Con and PCN vs. PND comparisons. (B) Gene Ontology (GO) enrichment analysis of biological processes enriched in the PND vs. Con comparison. (C) Kyoto Encyclopedia of Genes and Genomes (KEGG) pathway enrichment analysis of pathways enriched in PND compared with controls. (D–J) Gene Set Enrichment Analysis (GSEA) plots showing the enrichment profiles of apoptosis (D), TNF signaling pathway (E), necroptosis (F), NOD-like receptor signaling pathway (G), Toll-like receptor signaling pathway (H), IL-17 signaling pathway (I), and inflammatory mediator regulation of TRP channels (J). (K, L) Representative western blot images and quantitative analysis of pAKT and total AKT expression in hippocampal tissues. Data are presented as means **±** SD in Figure S12K (*n* = 3). Statistical significance was determined by one-way ANOVA followed by Tukey’s post hoc test. **P* <0.05, ****P* <0.001.

**Figure S13.** **Western blot quantification of glycolysis-related enzymes and microglial phenotype markers in BV2 cells.** Data are presented as means **±** SD (*n* = 3 independent experiments). Statistical significance was determined by one-way ANOVA followed by Tukey’s post hoc test. **P* <0.05, ***P* <0.01, ****P* <0.001, *****P* <0.0001.

**References**

[1]	HUANG L, TIAN W, CHEN X, et al. Peripheral Neutrophils-Derived Matrix Metallopeptidase-9 Induces Postoperative Cognitive Dysfunction in Aged Mice [J]. 2022, 14: 683295.
